# Prediction model for aortic dissection, aortic aneurysm, and peripheral artery disease

**DOI:** 10.1038/s41440-026-02730-5

**Published:** 2026-07-14

**Authors:** Yuta Suzuki, Hidehiro Kaneko, Akira Okada, Toshiyuki Ko, Takahiro Jimba, Atsushi Mizuno, Katsuhito Fujiu, Hiroyuki Morita, Norifumi Takeda, Koichi Node, Hideo Yasunaga, Norihiko Takeda

**Affiliations:** 1 Department of Cardiovascular Medicine, The University of Tokyo, Tokyo, Japan; 2 Center for Outcomes Research and Economic Evaluation for Health, National Institute of Public Health, Saitama, Japan; 3 Department of Advanced Cardiology, The University of Tokyo, Tokyo, Japan; 4 Department of Prevention of Diabetes and Lifestyle-Related Diseases, Graduate School of Medicine, The University of Tokyo, Tokyo, Japan; 5 Department of Cardiovascular Medicine, St. Luke’s International Hospital, Tokyo, Japan; 6 International University of Health and Welfare, Tokyo, Japan; 7 Department of Cardiovascular Medicine, Saga University, Saga, Japan; 8 Department of Clinical Epidemiology and Health Economics, School of Public Health, The University of Tokyo, Tokyo, Japan

**Keywords:** Risk prediction model, Aortic dissection, Aortic aneurysm, Peripheral artery disease, Implementation hypertension

## Abstract

Aortic dissection (AD), aortic aneurysm (AA), and peripheral artery disease (PAD) are associated with higher mortality. However, validated prediction models for these conditions remain scarce. We developed risk prediction models for AD, AA, and PAD using routine health check-up data. We used data from the DeSC database, encompassing 1,082,369 participants aged 20–74 years without prior cardiovascular disease for model derivation. Study participants were randomly split into derivation (50%) and internal validation (50%) cohorts. The primary outcomes were the incidence of AD, AA, and PAD. Flexible parametric survival models were used to estimate 5-year risk using routine health check-up data, including age, sex, body mass index, blood pressure, lipid profile, glucose, smoking status, physical activity, and medication use. During follow-up, 756 AD, 2,230 AA, and 4,131 PAD events occurred. In the internal validation cohort, Harrell’s C-index was 0.781 (95% confidence interval: 0.761 to 0.801) for AD, 0.812 (0.799 to 0.825) for AA, and 0.701 (0.690 to 0.711) for PAD. The Royston D statistic was 1.695 (1.533 to 1.857) for AD, 1.975 (1.874 to 2.075) for AA, and 1.222 (1.152 to 1.292) for PAD. The models had good calibration for each outcome. Risk prediction equations were developed and validated to estimate risk for the development of AD, AA, and PAD based on routine health check-up data. These models may allow risk stratification to support earlier diagnosis and intervention for vascular disease. Further external validation using datasets from different target populations and clinical settings is warranted.

**Figure:**
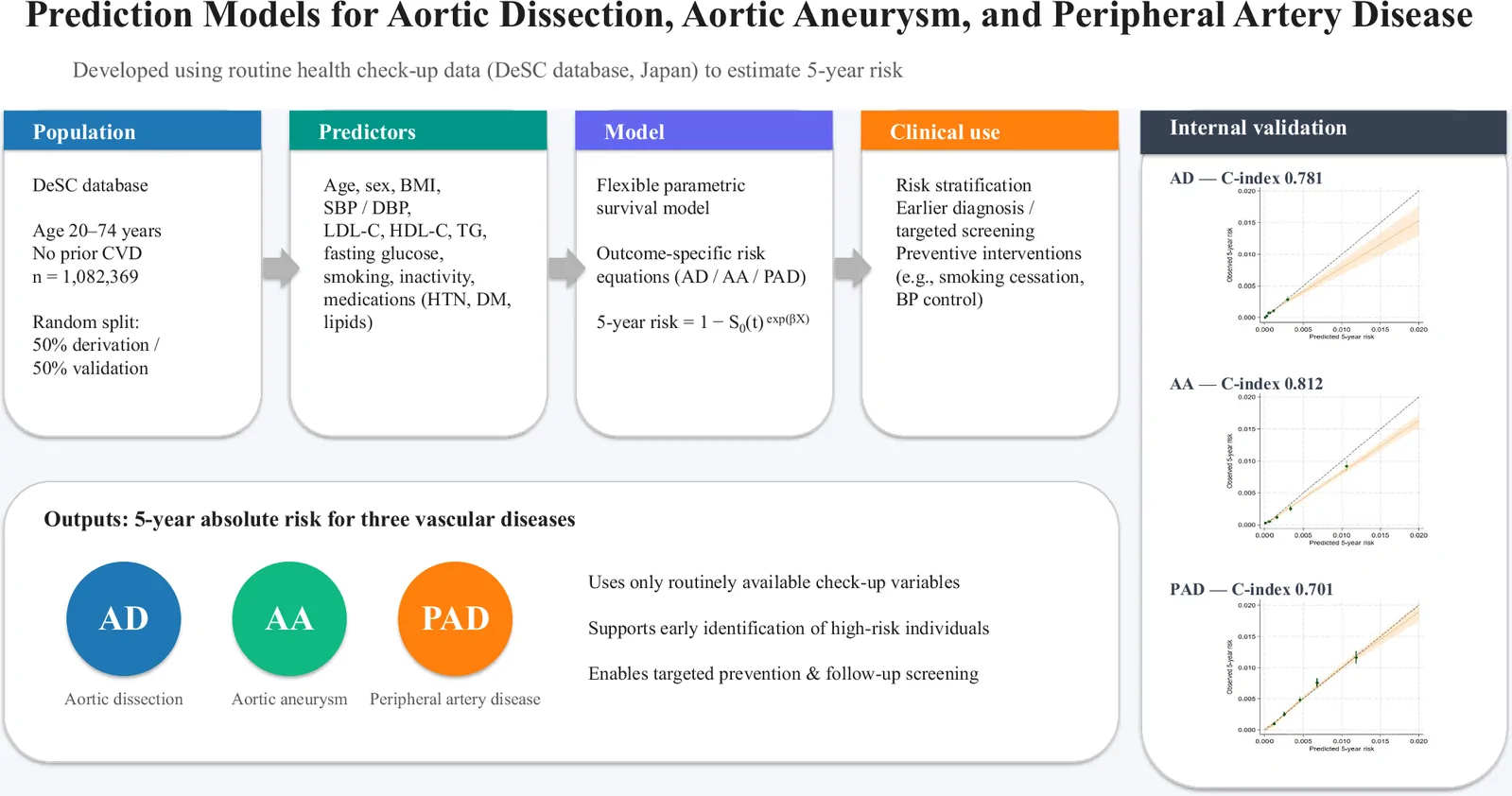

## Introduction

Cardiovascular risk prediction models such as the Framingham Risk Score, ASCVD risk estimator, and the Hisayama Study Score have been widely utilized for estimating the risk of coronary artery disease and stroke [1–5**]**. However, comparatively less attention has been paid to the prediction of other vascular diseases, including aortic dissection (AD), aortic aneurysm (AA), and peripheral artery disease (PAD). These conditions, although less prevalent, are associated with high morbidity and mortality [6–13], and their early identification remains a critical challenge in preventive cardiovascular medicine. Despite their clinical importance, there is a paucity of validated, population-based risk models specifically designed to predict the incidence of such vascular diseases using routine health checkup data. Leveraging large-scale real-world data that integrates health checkup data with administrative claims records, particularly in a nationally representative cohort, may offer a powerful tool to fill this gap. In this study, we developed and validated a simple and practical prediction model for AD, AA, and PAD using a nationwide health checkup and claims database. Our model is based solely on routinely available variables, allowing for broad implementation in clinical and occupational health settings. This work aims to contribute to the advancement of vascular risk stratification and population-based prevention strategies.

Point of view

- **Clinical relevance**
  Risk prediction equations for estimating the risk of AD, AA, and PAD using routine health check-up data may enable risk stratification to support earlier diagnosis and intervention.
- **Future direction**
  External validation in datasets derived from different target populations and clinical settings is warranted to assess model transportability.
- **Consideration for the Asian population**
  Because the prediction models were developed using variables collected through the Japanese standardized health checkup program, they may have applicability in countries with similar health checkup systems.

## Materials and methods

### Study design and population

This retrospective observational cohort study used the DeSC database, a large-scale administrative claims database in Japan [14].

The DeSC database consolidates extensive health checkup records with administrative claims data. It encompasses data from three types of Japanese insurance spanning from April 2014 to August 2023: (i) association/union-administered health insurance for salaried employees working for relatively large companies in Japan; (ii) National Health Insurance for unemployed individuals aged < 75 years; and (iii) the Advanced Elderly Medical Service System for older individuals aged ≥ 75 years. The DeSC database is noted for its extensive coverage and reliability, capturing a broad demographic spectrum in Japan, ranging from younger to older individuals [14, 15]. The database contains anonymized and aggregated patient data from both inpatient and outpatient services, facilitating longitudinal health tracking across multiple medical facilities. Diagnoses in the database are recorded using ICD-10 codes, and the database includes regular health checkup data such as laboratory results, body measurements, and lifestyle questionnaires.

We included 1,209,902 individuals aged 20–74 years who had available health check-up data for more than six months after insurance enrollment (a six-month look-back period). We excluded individuals with a prior history of cardiovascular disease (*n* = 127,308) and prior history of kidney replacement therapy (*n* = 225). The final study population from the DeSC database (*n* = 1,082,369) was randomly split into a derivation cohort (*n* = 541,185) and an internal validation cohort (*n* = 541,184) (Supplementary Fig. 1).

### Ethics

This research was approved by the ethical committee at the University of Tokyo under the approval numbers 2021010NI, following the principles of the Declaration of Helsinki. Given that all data utilized were anonymized, obtaining informed consent from participants was not required. Access to the DeSC database is granted to individuals who have procured it from DeSC Healthcare Inc.

### Predictors and outcomes

Clinical members of the study team proposed candidate predictors by their clinical expertise, given both traditional cardiovascular risk factors and findings from studies evaluating associations between routinely measured health check-up variables and outcomes (i.e., AD, AA, PAD) [16**–**18]. We included age, sex, body mass index (BMI), systolic and diastolic blood pressure, fasting plasma glucose, low-density lipoprotein cholesterol, high-density lipoprotein cholesterol, triglycerides, cigarette smoking status, physical inactivity, and the use of antihypertensive, antidiabetic, and lipid-lowering medications. We collected data regarding smoking habits (current or noncurrent) and physical activity (active or inactive) via self-administered questionnaires during the health check-up. From the administrative claims data, we identified prescription status for the following three classes of medications using the World Health Organization Anatomical Therapeutic Chemical (WHO-ATC) classification system: antihypertensive medications (ATC codes C02, C03, C04, C07, C08, and C09), antidiabetic medications (ATC code A10), and lipid-lowering medications (ATC code C10).

The primary outcomes were the incidence of AD, AA, and PAD. These were defined using International Classification of Diseases, 10th Revision (ICD-10) codes: I710 for AD; I711-716, I718, and I719 for AA; and I7020, I7021 for PAD. Outcome occurrence was defined as the first recorded diagnosis of the relevant ICD-10 code in the claims data. In the primary analysis, outcome ascertainment was based on ICD-10 codes alone, without reference to imaging or procedural records. Study participants were followed up from the index date (i.e., the initial health check-up) until the outcome occurrence, loss of insurance coverage (e.g., change of insurance, relocation, or death), or the study end date (August 2023).

### Statistical analysis

We used flexible parametric survival models to estimate the 5-year risk of each outcome in the derivation cohort. Flexible parametric survival models can accommodate right-censored survival data and estimate survival probabilities at prespecified time points under the assumption of non-informative censoring. Because these models estimate the baseline cumulative hazard function using cubic spline terms, they provide smooth estimates of the cumulative hazard function [19**–**21]. All candidate predictors were incorporated into the models, and coefficients for each predictor were estimated using the Stata command “stpm3”[22]. To examine whether categorizing continuous predictors improved model fit, we developed prediction models in which continuous predictors were entered as categorical variables, with thresholds determined by clinical cutoff values. Because categorization did not yield a superior model fit relative to the linear specification for any variable examined (as assessed by the Akaike information criterion, Bayesian information criterion, and likelihood ratio tests), continuous predictors were retained in their linear form in the final models.

The risk equation for predicting an individual’s 5-year risk of each outcome was then constructed based on the derivation cohort. All continuous variables were fitted after subtracting their reference values from each observed value. We estimated the 5-year baseline survival function in conjunction with the regression coefficients (β) and individual predictor values (X), resulting in the following formulation for predicted absolute risk over time [23, 24]:

$${{\rm{Predicted}}}\ 5{\mbox{-}}{{\rm{year}}}\ {{\rm{ risk}}}=1-{{{\rm{S}}}}_{0}{\left({{\rm{t}}}\right)}^{\exp \left({{\beta }}{{\rm{X}}}\right)}$$

We estimated the 5-year baseline survival function (S_0_[t = 5]) by setting all continuous variables and binary predictors to zero. The term βX represents the linear predictor.

Model performance of the final developed model equation was assessed in the derivation and internal validation cohorts. We evaluated standard statistical indices of model performance and discrimination, including Harrell’s C-index, the Royston D statistic, R^2^, and the calibration slope [25]. Calibration performance was further evaluated graphically in the internal validation cohort. Given the small number of events, participants were stratified into quintiles of predicted 5-year risk, and the mean predicted risk was plotted against the corresponding observed 5-year risk. Observed 5-year risk was estimated using the Kaplan-Meier method. To construct smooth calibration curves across risk groups, we employed pseudo-observations with a running non-parametric smoother.

We conducted five sensitivity analyses. First, we performed a temporal split validation as an alternative internal validation approach. Participants with an index date between 2014 and 2018 were assigned to the derivation sample, and those with an index date between 2019 and 2022 were assigned to the internal validation sample. The full model development procedure was repeated in the derivation sample (*n* = 691,012), and model performance was assessed in the temporal validation sample (*n* = 391,357). Second, to estimate the optimism of model performance, we performed bootstrap internal validation. We drew 200 bootstrap samples with replacements from the original dataset (i.e., overall study participants). Harrell’s C-index was evaluated in each bootstrap sample (bootstrap performance) and in the original dataset using the bootstrap-derived model (test performance). Optimism was calculated as the difference between bootstrap performance and test performance, averaged across 200 iterations. The optimism-corrected C-index was obtained by subtracting the mean optimism from the apparent C-index of the final model developed on the original dataset [26]. Third, we performed a geographical split validation. Participants were stratified by geographic region, defined according to the location of the health insurer, into eastern Japan (Hokkaido, Tohoku, Kanto and Koshin, and Hokuriku; *n* = 580,351) and western Japan (Tokai, Kinki, Chugoku, Shikoku, Kyushu, and Okinawa; *n* = 502,018). Model development was conducted in the eastern cohort, and validation was performed in the western cohort. Fourth, incident AD, AA, and PAD were each redefined as having at least two diagnosis codes recorded within a 6-month period, rather than a single recorded diagnosis. Fifth, incident AD, AA, and PAD were redefined as cases in which the ICD-10 diagnosis code was accompanied by hospitalization, diagnostic imaging, or therapeutic intervention. Therapeutic interventions were defined as aneurysmectomy or stent-graft insertion for AD and AA, and revascularization or limb amputation for PAD.

No formal sample size calculation was performed, and all available data were used for the analysis. Because we included only participants without missing data on any predictor, complete case analysis was performed. Statistical analyses were conducted using Stata v19 (StataCorp LLC, College Station, TX, USA).

## Results

### Baseline characteristics

Baseline characteristics of the study population are summarized in Table 1. The derivation and internal validation cohorts (DeSC database) had a median age of 59 years, with 47.0–47.2% men. The prevalence of medication use in the derivation and internal validation samples was 19.6% for antihypertensive medications, 5.1% for antidiabetic medications, and 16.1% for lipid-lowering medications.

**Table 1:** Baseline Characteristics

	Derivation Sample	Internal Validation
	*n* = 541,185	*n* = 541,184
Age, years	59 (46-67)	59 (46-67)
Men, n (%)	254,434 (47.0)	255,330 (47.2)
Body Mass Index, kg/m^2^	22.5 (20.3-24.8)	22.5 (20.4-24.9)
Systolic Blood Pressure, mmHg	123 (112-135)	123 (112-135)
Diastolic Blood Pressure, mmHg	75 (67-82)	75 (67-82)
Cigarette Smoking, n (%)	97,009 (17.9)	97,509 (18.0)
Physical inactivity, n (%)	241,127 (44.6)	240,101 (44.4)
Fasting Plasma Glucose, mg/dL	93 (87-101)	93 (87-101)
Low-Density Lipoprotein Cholesterol, mg/dL	123 (103-145)	123 (103-145)
High-Density Lipoprotein Cholesterol, mg/dL	63 (52-75)	63 (52-75)
Triglycerides, mg/dL	88 (63-129)	89 (63-128)
Antihypertensive Medications, n (%)	106,064 (19.6)	105,963 (19.6)
Antidiabetic Medications, n (%)	27,383 (5.1)	27,563 (5.1)
Lipid-lowering Medications, n (%)	87,370 (16.1)	87,144 (16.1)

Values are shown as *n* (%) or median (interquartile range)

### Model development

In the derivation cohort, 372 cases of AD (0.1%), 1169 cases of AA (0.2%), and 2,025 cases of PAD (0.4%) were recorded during the median follow-up period of 3.7 (Q1–Q3: 1.8–5.7) years. The incidence rate for the entire follow-up period of AD, AA, and PAD was 1.83 (1.65–2.03), 5.76 (5.44–6.10), and 9.98 (9.56–10.43) per 10,000 person-years, respectively. Information on the number of events and the incidence rate in the internal validation cohort is presented in Supplementary Table 1. The flexible parametric survival models identified several significant predictors for each outcome (Table 2). Age was positively associated with all three outcomes. Cigarette smoking was a strong predictor for AD (β = 0.80), AA (β = 0.68), and PAD (β = 0.38). Male sex was significantly associated with a higher risk of AD and AA but showed a weaker association with PAD. Regression coefficients, reference values of continuous variables, and baseline survival functions for the 5-year risk equations are presented in Table 3. An Excel-based risk calculator for estimating the risk of AD, AA, and PAD from the predictors is provided in the Supplementary Material.

**Table 2:** Prediction Model β coefficients in Derivation Sample

Predictors	β coefficient (95% Confidence interval)		
	Aortic Dissection	Aortic Aneurysm	Peripheral Artery Disease
Age, years	0.06 (0.05 to 0.07)	0.11 (0.10 to 0.12)	0.06 (0.05 to 0.06)
Men	0.43 (0.19 to 0.67)	0.91 (0.76 to 1.05)	0.08 (-0.02 to 0.18)
Body Mass Index, kg/m^2^	0.07 (0.04 to 0.10)	0.02 (0.00 to 0.04)	-0.01 (-0.02 to 0.01)
Systolic Blood Pressure, mmHg	0.02 (0.01 to 0.02)	0.00 (0.00 to 0.01)	0.00 (0.00 to 0.01)
Diastolic Blood Pressure, mmHg	0.01 (0.01 to 0.01)	0.01 (0.01 to 0.01)	0.00 (-0.01 to 0.01)
Cigarette Smoking	0.80 (0.57 to 1.04)	0.68 (0.55 to 0.81)	0.38 (0.27 to 0.49)
Physical inactivity	0.04 (-0.17 to 0.24)	0.00 (-0.12 to 0.12)	0.13 (0.04 to 0.22)
Fasting Plasma Glucose, mg/dL	-0.01 (-0.01 to 0.00)	0.00 (0.00 to 0.00)	0.00 (0.00 to 0.01)
Low-Density Lipoprotein Cholesterol, mg/dL	0.00 (0.00 to 0.00)	0.00 (0.00 to 0.00)	0.00 (0.00 to 0.00)
High-Density Lipoprotein Cholesterol, mg/dL	-0.01 (-0.02 to 0.00)	-0.02 (-0.02 to -0.01)	0.00 (-0.01 to 0.00)
Triglycerides, mg/dL	0.00 (0.00 to 0.00)	0.00 (0.00 to 0.00)	0.00 (0.00 to 0.00)
Antihypertensive Medications	0.37 (0.13 to 0.60)	0.38 (0.25 to 0.51)	0.20 (0.10 to 0.31)
Antidiabetic Medications	-1.33 (-2.07 to -0.58)	-0.47 (-0.73 to -0.21)	0.58 (0.43 to 0.74)
Lipid-lowering Medications	-0.06 (-0.34 to 0.22)	0.20 (0.05 to 0.34)	0.13 (0.01 to 0.24)

All variables are simultaneously included in the model

**Table 3:** Equation of the 5-year Risk of Each Outcome

	Aortic Dissection	Aortic Aneurysm	Peripheral Artery Disease
β coefficient			
Age, years	0.061978	0.106717	0.056084
Men	0.431036	0.909625	0.082403
Body Mass Index, kg/m^2^	0.066241	0.021908	-0.00626
Systolic Blood Pressure, mmHg	0.018123	0.002448	0.004196
Diastolic Blood Pressure, mmHg	0.009499	0.008862	-0.00072
Cigarette Smoking	0.80449	0.678116	0.382133
Physical inactivity	0.035275	-0.00299	0.13165
Fasting Plasma Glucose, mg/dL	-0.00651	-0.00077	0.003516
Low-Density Lipoprotein Cholesterol, mg/dL	0.000489	0.000378	0.001333
High-Density Lipoprotein Cholesterol, mg/dL	-0.01078	-0.01846	-0.00404
Triglycerides, mg/dL	-0.0008	-0.00018	0.000176
Antihypertensive Medications	0.367714	0.379645	0.202261
Antidiabetic Medications	-1.32667	-0.46875	0.584669
Lipid-lowering Medications	-0.05708	0.196938	0.125459
Reference Values Used in Calculations (Continuous Variables)			
Age, years	20		
Body Mass Index, kg/m^2^	22		
Systolic Blood Pressure, mmHg	120		
Diastolic Blood Pressure, mmHg	80		
Fasting Plasma Glucose, mg/dL	90		
Low-Density Lipoprotein Cholesterol, mg/dL	100		
High-Density Lipoprotein Cholesterol, mg/dL	60		
Triglycerides, mg/dL	100		
Risk Calculation			
5-year Risk, %	(1 - 0.999959^exp[βX]^)×100	(1 - 0.999984^exp[βX]^)×100	(1 - 0.999613^exp[βX]^)×100

All variables are simultaneously included in the model. All continuous variables were fitted after subtracting their reference values from each observed value. Binary variables were coded as 0 if absent and 1 if present. The estimated 5-year baseline survival functions were 0.999959 for aortic dissection, 0.999984 for aortic aneurysm, and 0.999613 for peripheral artery disease

### Model performance

Table 4 shows model performance in derivation and internal validation cohorts. The model demonstrated good discrimination for AD, with C-indices of 0.783 (0.761–0.805) and 0.781 (0.761–0.801) in the derivation and internal validation cohorts, respectively. Model performance based on the C-index was highest for AA, with values of 0.816 (0.804–0.827) and 0.812 (0.799–0.825) across the respective cohorts. Models provided moderate discrimination for PAD, with C-indices of 0.696 (0.684–0.707) and 0.701 (0.690–0.711) in the respective cohorts. The Royston D statistic in the internal validation cohort was 1.695 (1.533–1.857) for AD, 1.975 (1.874–2.075) for AA, and 1.222 (1.152–1.292) for PAD, respectively.

**Table 4:** Prediction Performance

	Apparent Performance in Derivation Cohort (95% Confidence Interval)	Performance in Internal Validation (95% Confidence Interval)
**Aortic Dissection**		
Harrell’s C index	0.783 (0.761 to 0.805)	0.781 (0.761 to 0.801)
Royston D statistic	1.751 (1.587 to 1.915)	1.695 (1.533 to 1.857)
R^2^	0.423 (0.375 to 0.467)	0.407 (0.359 to 0.452)
Calibration Slope	1.000 (0.910 to 1.090)	1.008 (0.907 to 1.108)
**Aortic Aneurysm**		
Harrell’s C index	0.816 (0.804 to 0.827)	0.812 (0.799 to 0.825)
Royston D statistic	2.002 (1.907 to 2.098)	1.975 (1.874 to 2.075)
R^2^	0.489 (0.465 to 0.512)	0.482 (0.456 to 0.507)
Calibration Slope	1.000 (0.946 to 1.054)	0.993 (0.935 to 1.051)
**Peripheral Artery Disease**		
Harrell’s C index	0.696 (0.684 to 0.707)	0.701 (0.690 to 0.711)
Royston D statistic	1.201 (1.130 to 1.272)	1.222 (1.152 to 1.292)
R^2^	0.256 (0.234 to 0.279)	0.263 (0.241 to 0.285)
Calibration Slope	1.000 (0.938 to 1.062)	1.030 (0.968 to 1.091)

Data are estimates (95% confidence intervals)

### Calibration

**Fig. 1:**
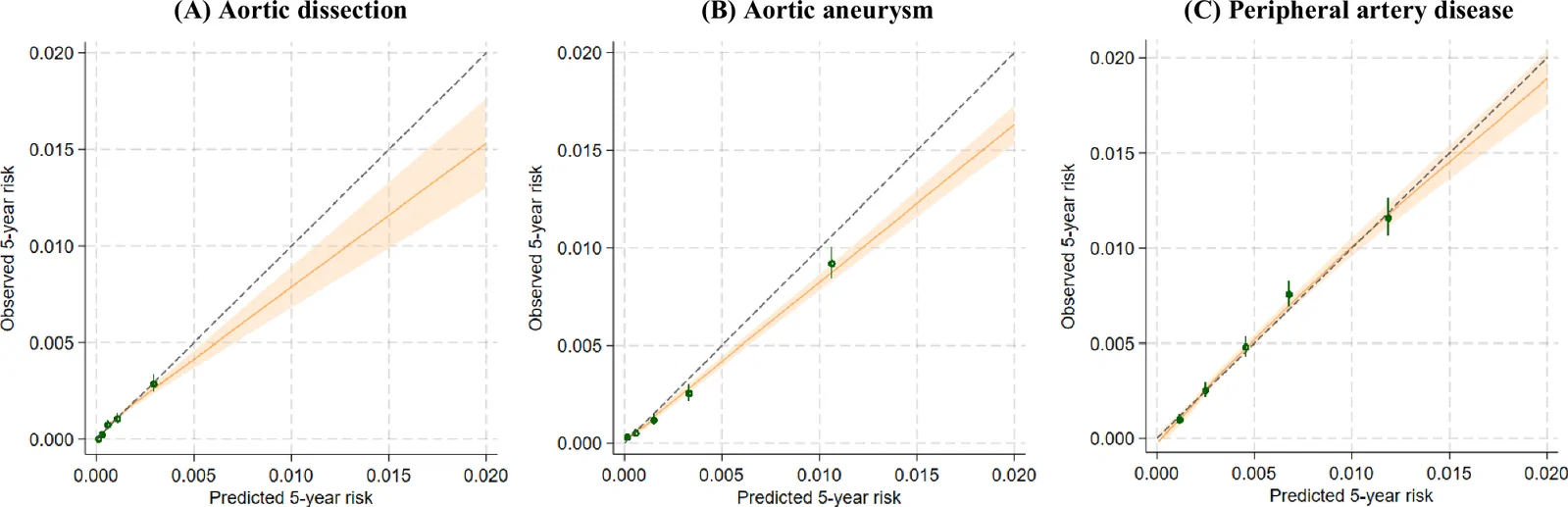
Calibration plots in the internal validation cohort. Calibration plots comparing the predicted versus observed 5-year risks for **A** aortic dissection, **B** aortic aneurysm, and **C** peripheral artery disease in the internal validation cohort. The x-axis represents the predicted 5-year risk calculated by the models, and the y-axis represents the observed 5-year risk. The dashed diagonal line indicates perfect calibration (ideal agreement between predicted and observed risks). The solid orange line represents the actual calibration of the model, with the shaded area indicating the 95% confidence interval. Green dots represent groups defined by quintiles of predicted risk, plotting mean predicted versus observed 5-year risk

Calibration slopes in the internal validation cohort were close to 1 for each outcome: 1.008 (0.907–1.108) for AD, 0.993 (0.935–1.051) for AA, and 1.030 (0.968–1.091) for PAD (Table 4). Calibration plots for the internal validation cohort visually showed a reasonable match between the predicted and observed 5-year risks for AD, AA, and PAD across quintiles of predicted risk (Fig. 1). The model showed slight risk overestimation around the 2% probability threshold for AD and AA, with the calibration curve falling marginally below perfect calibration.

### Sensitivity analysis

First, in the sensitivity analysis using temporal split validation, model coefficients were similar to those of the primary models across all three outcomes (Supplementary Table 2). The C-index for AD, AA, and PAD in the temporal validation sample was 0.751 (0.714–0.788), 0.812 (0.793–0.831), and 0.681 (0.661–0.700), respectively (Supplementary Table 3). Second, the model coefficients were similar to those of the primary models in the bootstrap internal validation (Supplementary Table 4). The mean optimism in the C-index was 0.0040 for AD, 0.0010 for AA, and 0.0007 for PAD (Supplementary Table 5). Third, in the sensitivity analysis using geographical split validation, model coefficients derived in eastern Japan were similar to those of the primary models across all three outcomes (Supplementary Table 6). The C-index for AD, AA, and PAD in the western Japan validation sample was 0.760 (0.738–0.781), 0.781 (0.768–0.794), and 0.655 (0.645–0.666), respectively (Supplementary Table 7). Fourth, incident AD, AA, and PAD were each redefined as having at least two ICD-10 diagnosis codes recorded within a 6-month period. Model performance, including discrimination and calibration, in the validation sample was similar to that observed in the primary analysis (Supplementary Table 8). Fifth, when outcomes were redefined to require accompanying hospitalization, diagnostic imaging, or therapeutic intervention in addition to the ICD-10 diagnosis code, model performance was similar to that of the primary analysis (Supplementary Table 9).

## Discussion

In this large-scale, nationwide study utilizing an integrated health checkup and administrative claims database, we developed and validated risk prediction models for AD, AA, and PAD using simple routine health examination data. To our knowledge, this is one of the first studies to construct and validate such models for these common life-threatening vascular diseases in a general population cohort. Our models demonstrated good to excellent discriminative ability (C-indices ranging from 0.701 to 0.812) and high calibration (slopes approximating 1.0) in the internal validation cohort, suggesting their potential utility for early identification and preventive intervention in clinical and public health settings. Notably, the prediction models for AD and AA achieved particularly high discrimination (C-index 0.781 and 0.812, respectively), while the PAD model showed moderate performance (C-index 0.701). These results suggest that standard cardiovascular risk factors collected in routine checkups—such as age, sex, blood pressure, and smoking status—are robust predictors for aortic pathologies in the Japanese population.

AD and AA are often silent until catastrophic events occur. PAD, though more slowly progressive, is frequently underdiagnosed and can significantly impact quality of life and mortality [7,9,10]. Early identification of individuals at elevated risk offers a unique opportunity for preventive measures such as lifestyle modification, smoking cessation support, tighter blood pressure control, and in some cases, further diagnostic imaging [27**–**29]. Previous risk models have predominantly focused on coronary artery disease and stroke, supported by landmark cohorts such as the Framingham Heart Study, the ACC/AHA ASCVD pooled cohort equations, and the Hisayama Study (a Japanese regional cohort) Score [1, 4, 30]. These models have guided clinical decision-making and public health strategies for decades from the perspective of CVD prevention, particularly for ischemic heart disease and stroke [31]. In contrast, fewer efforts have been made to develop prediction tools for the aforementioned vascular diseases, despite their high morbidity and mortality once clinical events occur. Several prediction models for abdominal AA have been proposed and include predictors broadly consistent with those identified in our study, such as age, sex, smoking status, blood pressure, and lipid-related variables [32, 33]. However, many of these models were developed specifically for abdominal AA and often rely on predictors that are not routinely available in standard health checkup settings, including family history of abdominal AA, detailed dietary information or intermittent claudication, thereby limiting their broader applicability in population-based screening strategies. Some studies have proposed risk markers or screening strategies for abdominal AA, such as the U.S. Preventive Services Task Force (USPSTF) recommendation for one-time ultrasound screening in older male smokers [34, 35]. However, no well-established multivariable prediction model using generalizable population-level data and routine clinical variables exists for AD, AA, or PAD to inform screening strategies for these conditions.

Our study addresses this important clinical gap by demonstrating that even basic anthropometric and laboratory parameters—when analyzed through modern statistical methods—can yield useful risk stratification tools for these vascular diseases. The models we present are designed to be simple and practical, utilizing only variables typically available in the standardized health checkup program in Japan. This feature allows for seamless integration into existing health infrastructure, minimizing the cost and effort of implementation. Moreover, risk scores derived from our models could be used to inform shared decision-making during annual health checkups, targeting high-risk individuals for further screening or clinical follow-up. Further, from a public health perspective, our findings illustrate the potential of real-world data in identifying at-risk subpopulations for vascular diseases that would be difficult to study prospectively. By applying advanced data science methodologies to a large-scale dataset, we demonstrate the feasibility of using preventive health data not only for screening of metabolic syndrome or cancer, but also for prediction of serious vascular events.

### Strengths of the study

Several features strengthen the validity and applicability of our findings. First, for model development, we used a large, nationally representative dataset combining health checkup results with longitudinal claims data, encompassing a diverse population across age, sex, and clinical backgrounds. Second, our models were built solely on data from standard health checkups, without requiring additional imaging, genetic data, or specialized biomarkers. This simplicity enhances the scalability and practicality of our approach, making it applicable not only in Japan, but potentially in other countries with similar screening systems. Finally, we provided outcome-specific models, allowing for targeted prediction and interpretation. This is particularly important given the distinct pathophysiology and clinical presentation of AD, AA, and PAD, which may not be adequately captured by general cardiovascular risk scores. Indeed, higher fasting plasma glucose levels were inversely associated with the risk of AD and AA in our models. This inverse association may reflect hyperglycemia-related suppression of metalloproteinase activity and reduced adventitial neovascularization [16, 17, 36–38]. These mechanisms may in turn reduce vascular smooth muscle cell death and extracellular matrix degradation, thereby attenuating the development of AD and AA. Nevertheless, clinicians may counsel patients that optimal glycemic control remains important for overall cardiovascular risk reduction, given that diabetes substantially increases the risk of other cardiovascular diseases.

### Future directions

The present study opens several avenues for further investigation. First, prospective studies could evaluate whether integrating these risk scores into routine checkups improves detection rates or outcomes through earlier diagnosis and intervention. Second, combining our models with imaging-based screening tools (e.g., point-of-care ultrasound) in high-risk individuals may enhance diagnostic efficiency. Third, incorporating additional information such as family history, more detailed prescription data, or social determinants of health may further refine risk estimates. Notably, the predictive performance of our models was superior for AD and AA relative to PAD. In particular, the superior discriminative performance for AD and AA (C-statistics 0.781 and 0.812, respectively) compared to PAD (C-statistic 0.701) in the internal validation cohort may reflect differing etiologies. AD and AA share strong pathophysiological links with connective tissue integrity and hemodynamic stress, which are closely captured by core variables like age, blood pressure, and smoking. In contrast, PAD is a more heterogeneous endpoint of systemic atherosclerosis, influenced by a broader array of factors not fully captured in our models (e.g., inflammation, microvascular disease). This suggests that while our model is highly effective for aortic-centric pathologies, predicting generalized atherosclerosis manifestations may require additional predictors. Finally, the calibration of our models may not be perfect. The calibration slope indicated some overestimation around the 2% predicted risk threshold for AD and AA. However, the magnitude of this overestimation was marginal. In addition, given that individuals within this risk range would generally be considered at relatively higher risk regardless of small differences in predicted probabilities, the potential impact on clinical interpretation and decision-making is likely to be minimal.

#### Perspective of Asia

Abdominal AA screening has been implemented in the United States and several European countries [39**–**41]. In contrast, population-based AA screening may be less widely implemented in Asian countries, including Japan [42, 43]. The prediction models developed in the present study use routine health check-up data and may support the development of screening strategies in Asian populations.

### Limitations

Our study has several limitations. First, the diagnoses of AD, AA, and PAD relied on ICD-10 codes recorded in administrative claims databases. Although the validity of diagnostic codes for cardiovascular diseases recorded in Japanese administrative databases has been examined in several studies [44**–**47], it should be noted that the diagnoses recorded in claims databases such as the DeSC database have not been fully validated. For example, a recent study in Japan validated ICD-10 codes for AD in the Diagnosis Procedure Combination database and reported a sensitivity of 100% and a specificity of 96.6% [46]. Furthermore, combining ICD-10 codes with hospitalization status, procedural data, and diagnostic imaging has been shown to achieve acceptable sensitivity and positive predictive value for acute aortic disease in Japanese health insurance claims data [47]. However, asymptomatic cases and events occurring outside the hospital setting may not be fully captured in claims-based databases, potentially leading to underestimation of true outcome incidence and bias in outcome ascertainment. Second, in the present study, the temporal relationship between diagnosis and death could not be determined with sufficient granularity. Therefore, deaths due to aortic dissection occurring before hospital arrival, including dead-on-arrival cases, could not be specifically identified. This represents a limitation of the present study and may have led to underascertainment of the most severe cases. A population-based study in Japan reported that among patients with acute aortic dissection, 60.8% of those with type A and 21.4% of those with type B died before reaching the hospital [48]. Third, our dataset lacked detailed clinical information that could influence risk, such as aneurysm diameter, specific genetic factors, family history of aortic diseases, or the duration of smoking. Fourth, the relatively short follow-up period of our dataset might have introduced uncertainty in the estimated risks. Finally, the models in this study underwent only internal validation. Because the study population consisted exclusively of Japanese individuals, the predictive performance of our models in other ethnic groups remains unknown. Furthermore, as the study population was restricted to individuals with available health checkup data in the DeSC database, selection bias cannot be ruled out. External validation using cohorts from different countries, regions, and ethnic populations is essential to establish model transportability. Future studies should conduct such validation in populations with diverse ethnic and genetic backgrounds, as well as varying baseline cardiovascular risk profiles, to assess model performance and applicability across different healthcare settings and geographical contexts.

## Conclusions

We developed and validated prognostic models for predicting the development of AD, AA, and PAD using data from routine health check-ups. These models may allow for risk stratification to support earlier diagnosis and intervention and may provide information to guide individualized clinical decision-making. Further external validation across different target populations and clinical settings is warranted.

## Supplementary information

Supplementary Tables

Supplementary Figure 1

Supplementary Material

Supplementary Figure 1 legends

## Data Availability

The DeSC database is available for purchase from DeSC Healthcare Inc.
