# Supplementary material for: Prediction model for aortic dissection, aortic aneurysm, and peripheral artery disease
Source: Hypertens Res. 2026 Jul 14;49(9):2499–509. doi: 10.1038/s41440-026-02730-5 (PMC13547102; doi:10.1038/s41440-026-02730-5)

Supplementary Figure 1. Flowchart of the study population for model derivation and internal validation

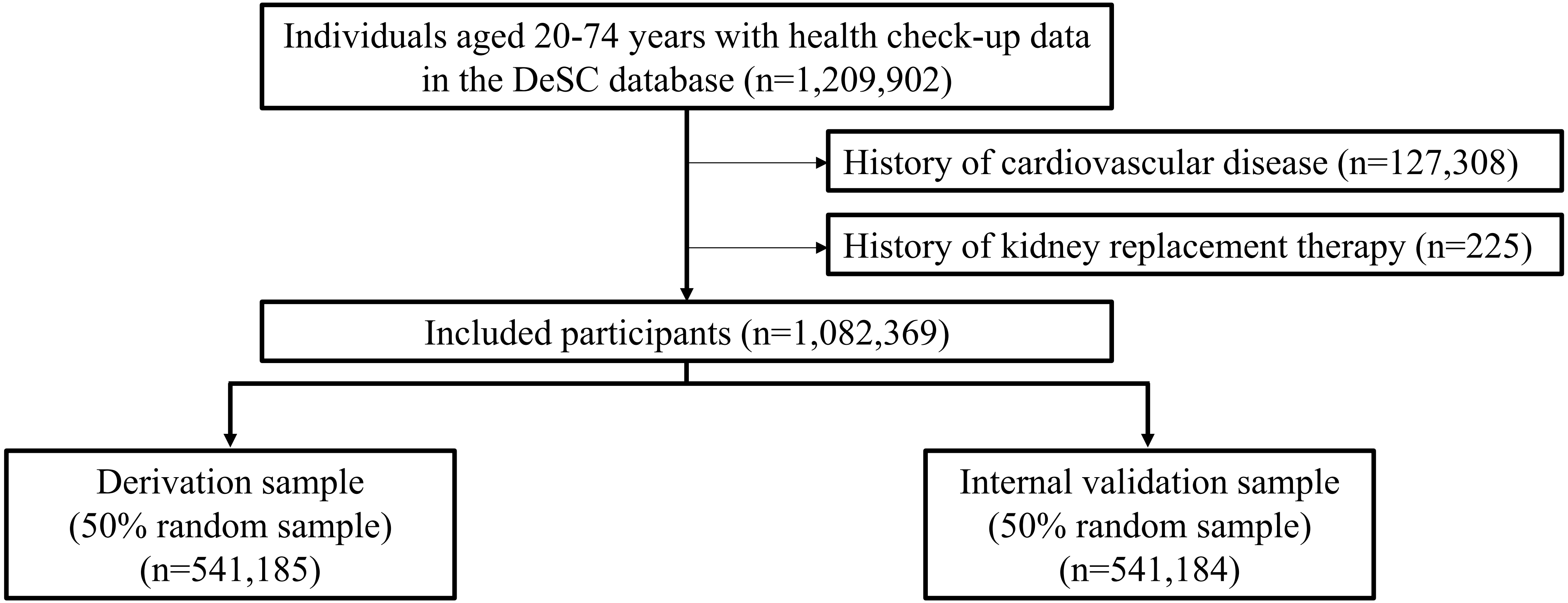

Supplement: Supplementary file 1 [file 41440_2026_2730_MOESM2_ESM.pdf]
