# Supplementary material for: Prediction model for aortic dissection, aortic aneurysm, and peripheral artery disease
Source: Hypertens Res. 2026 Jul 14;49(9):2499–509. doi: 10.1038/s41440-026-02730-5 (PMC13547102; doi:10.1038/s41440-026-02730-5)
Supplement: Supplementary file 2 [file 41440_2026_2730_MOESM3_ESM.pdf]

Note for worksheet: Please enter values in all highlighted blue cells. Model included age, sex, body mass index, systolic and diastolic blood pressure, fasting plasma glucose, low-density lipoprotein cholesterol, high-density lipoprotein cholesterol, triglycerides, cigarette smoking status, physical inactivity, and the use of antihypertensive, antidiabetic, and lipid-lowering medications. After entering all predictor values, the predicted 5-year risk will be displayed below.

| **Predictor** | Value |
| --- | --- |
| Age (20-74 years) | 65 |
| Sex (men=1, women=0) | 1 |
| Body mass index (kg/m$^2$) | 25 |
| Systolic blood pressure (mmHg) | 180 |
| Diastolic blood pressure (mmHg) | 120 |
| Cigarette Smoking (current=1, non-current=0) | 1 |
| Physical inactivity (inactive=1, active=0) | 1 |
| Fasting plasma glucose (mg/dL) | 100 |
| Low-density lipoprotein cholesterol (mg/dL) | 150 |
| High-density lipoprotein cholesterol (mg/dL) | 50 |
| Triglycerides (mg/dL) | 150 |
| Antihypertensive medications (with=1, without=0) | 1 |
| Antidiabetic medications (with=1, without=0) | 0 |
| Lipid-lowering medications (with=1, without=0) | 0 |

| **Predicted 5-year risk (%)** | |
| --- | --- |
| Aortic dissection | 1.85 |
| Aortic aneurysm | 2.91 |
| Peripheral artery disease | 1.52 |
