# Supplementary material for: Prediction model for aortic dissection, aortic aneurysm, and peripheral artery disease
Source: Hypertens Res. 2026 Jul 14;49(9):2499–509. doi: 10.1038/s41440-026-02730-5 (PMC13547102; doi:10.1038/s41440-026-02730-5)
Supplement: Supplementary file 3 [file 41440_2026_2730_MOESM1_ESM.docx]

| **Supplementary Table 1. Number and Incidence Rate of Each Outcome** | | |
| --- | --- | --- |
|  | Derivation Sample | Internal Validation |
|  | n=541,185 | n=541,184 |
| Follow-up Years, median (Q1-Q3) | 3.7 (1.8-5.7) | 3.7 (1.8-5.7) |
| **Aortic Dissection** |  |  |
| Number of Events, n (%) | 372 (0.1) | 384 (0.1) |
| Incidence Rate, Point Estimate (95% Confidence Interval) | 1.83 (1.65 to 2.03) | 1.89 (1.71 to 2.09) |
| **Aortic Aneurysm** |  |  |
| Number of Events, n (%) | 1,169 (0.2) | 1,061 (0.2) |
| Incidence Rate, Point Estimate (95% Confidence Interval) | 5.76 (5.44 to 6.10) | 5.24 (4.93 to 5.56) |
| **Peripheral Artery Disease** |  |  |
| Number of Events, n (%) | 2,025 (0.4) | 2,106 (0.4) |
| Incidence Rate, Point Estimate (95% Confidence Interval) | 9.98 (9.56 to 10.43) | 10.41 (9.98 to 10.87) |
| The incidence rate was per 10,000 person-years. Values were calculated for the entire follow-up period in each sample. | | |

| **Supplementary Table 2. Equation of the 5-year Risk of Each Outcome in the Temporal Split Validation** | | | |  |
| --- | --- | --- | --- | --- |
|  | Aortic Dissection | Aortic Aneurysm | Peripheral Artery Disease |  |
| β coefficient | | | |  |
| Age, years | 0.073639 | 0.111658 | 0.061268 |  |
| Men | 0.440539 | 0.944688 | 0.130512 |  |
| Body Mass Index, kg/m^2^ | 0.044410 | 0.007953 | -0.011386 |  |
| Systolic Blood Pressure, mmHg | 0.013824 | -0.001112 | 0.005567 |  |
| Diastolic Blood Pressure, mmHg | 0.016360 | 0.014368 | -0.003062 |  |
| Cigarette Smoking | 0.940692 | 0.712275 | 0.362214 |  |
| Physical inactivity | -0.000674 | 0.019730 | 0.142784 |  |
| Fasting Plasma Glucose, mg/dL | -0.008126 | -0.002135 | 0.004144 |  |
| Low-Density Lipoprotein Cholesterol, mg/dL | 0.000119 | 0.001957 | 0.002188 |  |
| High-Density Lipoprotein Cholesterol, mg/dL | -0.013009 | -0.017047 | -0.000515 |  |
| Triglycerides, mg/dL | -0.000627 | -0.000274 | 0.000606 |  |
| Antihypertensive Medications | 0.405933 | 0.349944 | 0.160250 |  |
| Antidiabetic Medications | -0.614738 | -0.354522 | 0.535983 |  |
| Lipid-lowering Medications | -0.015479 | 0.162344 | 0.204045 |  |
| Risk Calculation | | | |  |
| 5-year Risk, % | (1 - 0.999973^exp[βX]^)×100 | (1 - 0.999988^exp[βX]^)×100 | (1 - 0.999699^exp[βX]^)×100 |  |
| All variables are simultaneously included in the model. All continuous variables were fitted after subtracting their reference values from each observed value. Binary variables were coded as 0 if absent and 1 if present. Reference values: age, 20 years; body mass index, 22 kg/m^2^; systolic blood pressure, 120 mmHg; diastolic blood pressure, 80 mmHg; fasting plasma glucose, 90 mg/dL; low-density lipoprotein cholesterol, 100 mg/dL; high-density lipoprotein cholesterol, 60 mg/dL; triglycerides, 100 mg/dL. | | | | |

| **Supplementary Table 3. Prediction Performance in the Temporal Split Validation** | | |
| --- | --- | --- |
|  | Apparent Performance in Derivation Cohort (95% Confidence Interval) | Performance in Internal Validation (95% Confidence Interval) |
| **Aortic Dissection** |  |  |
| Harrell’s C index | 0.797 (0.782 to 0.812) | 0.751 (0.714 to 0.788) |
| **Aortic Aneurysm** |  |  |
| Harrell’s C index | 0.814 (0.804 to 0.823) | 0.812 (0.793 to 0.831) |
| **Peripheral Artery Disease** |  |  |
| Harrell’s C index | 0.707 (0.699 to 0.715) | 0.681 (0.661 to 0.700) |
| Data are estimates (95% confidence intervals). | | |

| **Supplementary Table 4. Equation of the 5-year Risk of Each Outcome in the Bootstrap Internal Validation** | | | |  |
| --- | --- | --- | --- | --- |
|  | Aortic Dissection | Aortic Aneurysm | Peripheral Artery Disease |  |
| β coefficient | | | |  |
| Age, years | 0.070716 | 0.109238 | 0.058450 |  |
| Men | 0.442676 | 0.973023 | 0.135065 |  |
| Body Mass Index, kg/m^2^ | 0.053496 | 0.008344 | -0.009478 |  |
| Systolic Blood Pressure, mmHg | 0.015422 | 0.000886 | 0.0048917 |  |
| Diastolic Blood Pressure, mmHg | 0.010064 | 0.009476 | -0.002777 |  |
| Cigarette Smoking | 0.880237 | 0.715495 | 0.377353 |  |
| Physical inactivity | 0.067406 | 0.027335 | 0.148947 |  |
| Fasting Plasma Glucose, mg/dL | -0.006964 | -0.001934 | 0.0042846 |  |
| Low-Density Lipoprotein Cholesterol, mg/dL | 0.000638 | 0.0016472 | 0.002112 |  |
| High-Density Lipoprotein Cholesterol, mg/dL | -0.010229 | -0.016407 | -0.000000 |  |
| Triglycerides, mg/dL | -0.000722 | -0.000146 | 0.000601 |  |
| Antihypertensive Medications | 0.389059 | 0.367903 | 0.160495 |  |
| Antidiabetic Medications | -0.7042303 | -0.373201 | 0.515834 |  |
| Lipid-lowering Medications | -0.024729 | 0.188880 | 0.202407 |  |
| Risk Calculation | | | |  |
| 5-year Risk, % | (1 - 0.999971^exp[βX]^)×100 | (1 - 0.999987^exp[βX]^)×100 | (1 - 0.999679^exp[βX]^)×100 |  |
| All variables are simultaneously included in the model. All continuous variables were fitted after subtracting their reference values from each observed value. Binary variables were coded as 0 if absent and 1 if present. Reference values: age, 20 years; body mass index, 22 kg/m^2^; systolic blood pressure, 120 mmHg; diastolic blood pressure, 80 mmHg; fasting plasma glucose, 90 mg/dL; low-density lipoprotein cholesterol, 100 mg/dL; high-density lipoprotein cholesterol, 60 mg/dL; triglycerides, 100 mg/dL. | | | | |

| **Supplementary Table 5. Discrimination and Optimism Estimates from the Bootstrap Internal Validation** | | | |
| --- | --- | --- | --- |
|  | Apparent Performance (95% Confidence Interval) | Mean Optimism | Optimism-Corrected Performance |
| **Aortic Dissection** |  |  |  |
| Harrell’s C index | 0.7889 | 0.0040 | 0.7850 |
| **Aortic Aneurysm** |  |  |  |
| Harrell’s C index | 0.8184 | 0.0010 | 0.8174 |
| **Peripheral Artery Disease** |  |  |  |
| Harrell’s C index | 0.7047 | 0.0007 | 0.7040 |
| We drew 200 bootstrap samples with replacements from the original dataset. The full model development procedure was repeated in each bootstrap sample. Mean optimism was defined as the average difference between model performance evaluated in the bootstrap sample and performance of the same bootstrap-derived model evaluated in the original dataset. The optimism-corrected performance was obtained by subtracting the mean optimism from the apparent performance of the final model. | | | |

| **Supplementary Table 6. Equation of the 5-year Risk of Each Outcome in the Geographical Split Validation** | | | |  |
| --- | --- | --- | --- | --- |
|  | Aortic Dissection | Aortic Aneurysm | Peripheral Artery Disease |  |
| β coefficient | | | |  |
| Age, years | 0.064701 | 0.105651 | 0.051193 |  |
| Men | 0.499011 | 0.909501 | 0.089212 |  |
| Body Mass Index, kg/m^2^ | 0.043706 | 0.000980 | -0.013807 |  |
| Systolic Blood Pressure, mmHg | 0.019366 | 0.004175 | 0.001105 |  |
| Diastolic Blood Pressure, mmHg | 0.009278 | 0.008040 | 0.003889 |  |
| Cigarette Smoking | 0.871685 | 0.707602 | 0.291839 |  |
| Physical inactivity | -0.003789 | -0.021854 | 0.103730 |  |
| Fasting Plasma Glucose, mg/dL | -0.012521 | -0.002253 | 0.006639 |  |
| Low-Density Lipoprotein Cholesterol, mg/dL | 0.002283 | 0.001863 | 0.002619 |  |
| High-Density Lipoprotein Cholesterol, mg/dL | -0.010520 | -0.017920 | -0.000482 |  |
| Triglycerides, mg/dL | -0.000630 | 0.000228 | 0.000513 |  |
| Antihypertensive Medications | 0.394480 | 0.450127 | 0.177517 |  |
| Antidiabetic Medications | -1.033602 | -0.533309 | 0.578900 |  |
| Lipid-lowering Medications | 0.083450 | 0.190982 | 0.123072 |  |
| Risk Calculation | | | |  |
| 5-year Risk, % | (1 - 0.999967^exp[βX]^)×100 | (1 - 0.999985^exp[βX]^)×100 | (1 - 0.999613^exp[βX]^)×100 |  |
| All variables are simultaneously included in the model. All continuous variables were fitted after subtracting their reference values from each observed value. Binary variables were coded as 0 if absent and 1 if present. Reference values: age, 20 years; body mass index, 22 kg/m^2^; systolic blood pressure, 120 mmHg; diastolic blood pressure, 80 mmHg; fasting plasma glucose, 90 mg/dL; low-density lipoprotein cholesterol, 100 mg/dL; high-density lipoprotein cholesterol, 60 mg/dL; triglycerides, 100 mg/dL. | | | | |

| **Supplementary Table 7. Prediction Performance in the Geographical Split Validation** | | |
| --- | --- | --- |
|  | Apparent Performance in Derivation Cohort (95% Confidence Interval) | Performance in Internal Validation (95% Confidence Interval) |
| **Aortic Dissection** |  |  |
| Harrell’s C index | 0.793 (0.771 to 0.816) | 0.760 (0.738 to 0.781) |
| **Aortic Aneurysm** |  |  |
| Harrell’s C index | 0.835 (0.823 to 0.848) | 0.781 (0.768 to 0.794) |
| **Peripheral Artery Disease** |  |  |
| Harrell’s C index | 0.705 (0.693 to 0.717) | 0.655 (0.645 to 0.666) |
| Data are estimates (95% confidence intervals). | | |

| **Supplementary Table 8. Prediction Performance Using an Alternative Outcome Definition Requiring at Least Two Diagnosis Codes Within a 6-Month Period** | |
| --- | --- |
|  | Performance in Internal Validation (95% Confidence Interval) |
| **Aortic Dissection** |  |
| Harrell’s C index | 0.791 (0.767 to 0.815) |
| Royston D statistic | 1.800 (1.603 to 1.996) |
| R^2^ | 0.436 (0.380 to 0.487) |
| Calibration Slope | 1.074 (0.951 to 1.197) |
| **Aortic Aneurysm** |  |
| Harrell’s C index | 0.823 (0.809 to 0.838) |
| Royston D statistic | 2.066 (1.944 to 2.188) |
| R^2^ | 0.505 (0.474 to 0.533) |
| Calibration Slope | 1.064 (0.992 to 1.137) |
| **Peripheral Artery Disease** |  |
| Harrell’s C index | 0.713 (0.701 to 0.726) |
| Royston D statistic | 1.304 (1.219 to 1.388) |
| R^2^ | 0.289 (0.262 to 0.315) |
| Calibration Slope | 1.109 (1.033 to 1.184) |
| Data are estimates (95% confidence intervals). | |

| **Supplementary Table 9. Prediction Performance Using an Outcome Definition Requiring Hospitalization, Diagnostic Imaging, or Therapeutic Intervention** | |
| --- | --- |
|  | Performance in Internal Validation (95% Confidence Interval) |
| **Aortic Dissection** |  |
| Harrell’s C index | 0.780 (0.758 to 0.802) |
| Royston D statistic | 1.713 (1.539 to 1.886) |
| R^2^ | 0.412 (0.361 to 0.459) |
| Calibration Slope | 1.018 (0.910 to 1.126) |
| **Aortic Aneurysm** |  |
| Harrell’s C index | 0.812 (0.799 to 0.825) |
| Royston D statistic | 1.976 (1.875 to 2.078) |
| R^2^ | 0.483 (0.456 to 0.508) |
| Calibration Slope | 0.995 (0.936 to 1.053) |
| **Peripheral Artery Disease** |  |
| Harrell’s C index | 0.705 (0.695 to 0.716) |
| Royston D statistic | 1.255 (1.182 to 1.328) |
| R^2^ | 0.273 (0.250 to 0.296) |
| Calibration Slope | 1.063 (0.998 to 1.128) |
| Data are estimates (95% confidence intervals). | |
