# Supplementary material for: Prediction model for aortic dissection, aortic aneurysm, and peripheral artery disease
Source: Hypertens Res. 2026 Jul 14;49(9):2499–509. doi: 10.1038/s41440-026-02730-5 (PMC13547102; doi:10.1038/s41440-026-02730-5)
Supplement: Supplementary file 4 [file 41440_2026_2730_MOESM4_ESM.docx]

**Supplementary Figure 1. Flowchart of the study population for model derivation and internal validation.** Selection process of the study participants from the DeSC database. Individuals aged 20–74 years with available health check-up data were included. After excluding those with a history of cardiovascular disease or kidney replacement therapy, the final population (n=1,082,369) was randomly split into a derivation sample (n=541,185) and an internal validation sample (n=541,184).
